# Supplementary material for: Bromodomain-containing Protein 4 regulates innate inflammation via modulation of alternative splicing
Source: Front Immunol. 2023 Jun 26;14:1212770. doi: 10.3389/fimmu.2023.1212770 (PMC10331468; doi:10.3389/fimmu.2023.1212770)
Supplement: Supplementary file 1 [file DataSheet_1.zip › Supplementary Figures.pdf]

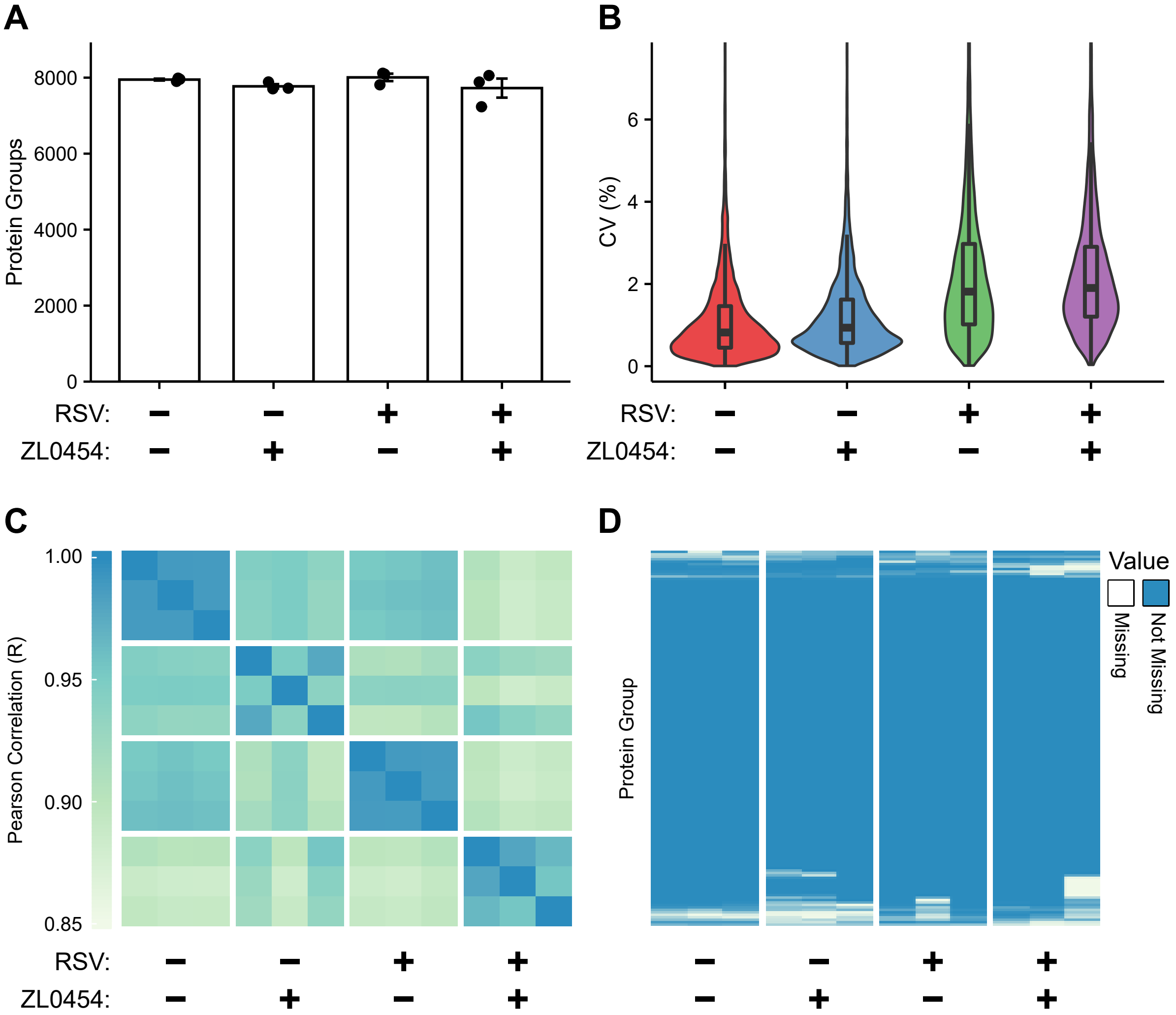

Figure S1: Quality control metrics for diaPASEF LCMS analysis. (A) Quantifiable proteins per experimental group. (B) Coefficient of Variance (%CV) violin plots indicating intra-group median variance to protein abundance measurements. (C) Heatmap indicating sample-to-sample Pearson Correlation scores. (D) Data completeness matrix of quantifiable proteins. Data is prior to filtering.

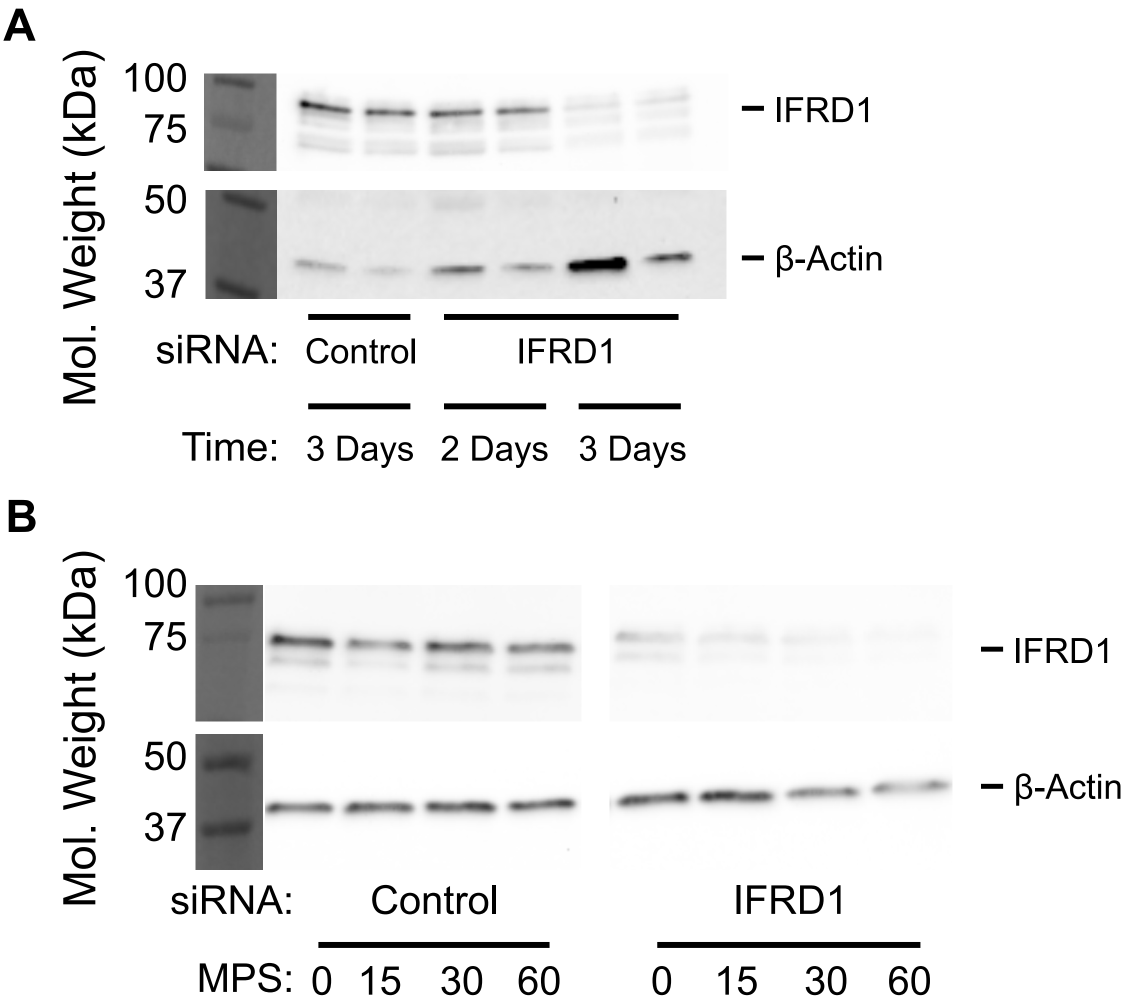

Figure S2: Optimization of IFRD1 knockdown by siRNA in hSAECs. (A) Western blot indicating peak protein knockdown occurs at 72+ hours. (B). Western blot demonstrating that IFRD1 protein abundance is stable throughout inflammatory activation by polyIC (0-60 minutes post-stimulation/MPS) after treatment with either non-targetting or IFRD1-specific siRNA.

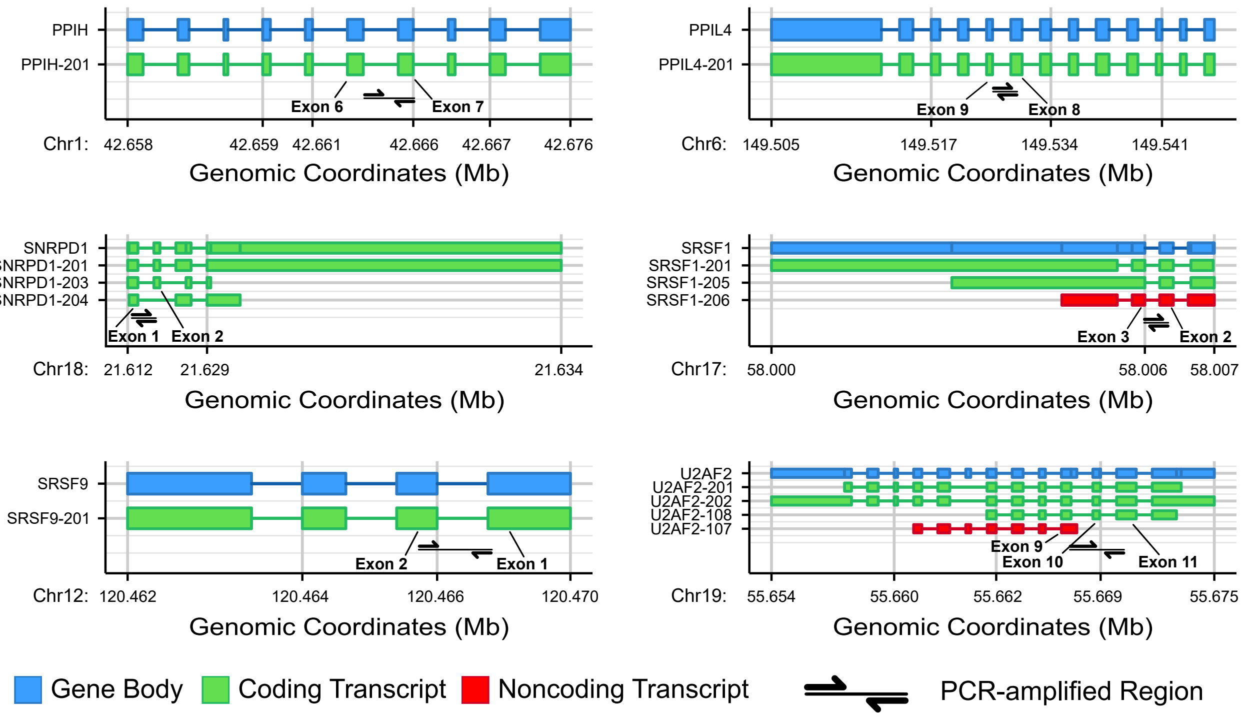

Figure S3: Schematic of spliceosome component transcripts and regions validated by q-RT-PCR. Mapped transcripts are plotted in compressed genomic coordinates and color coded according to gene body and protein-coding status.

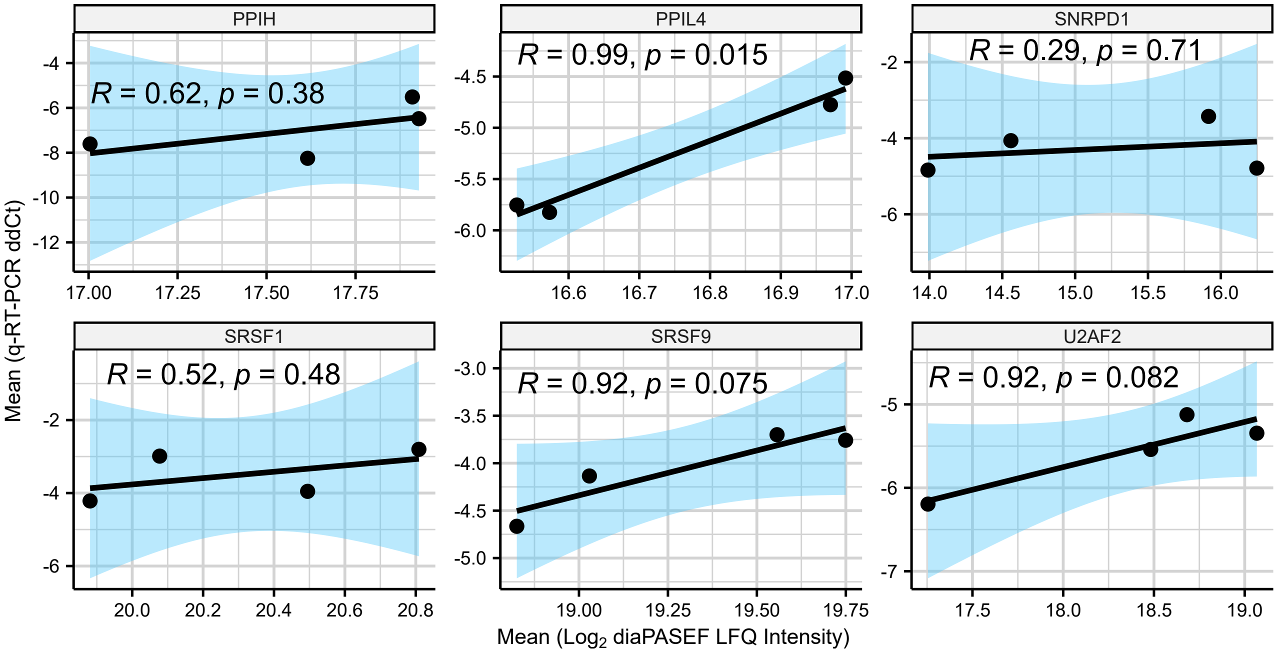

Figure S4: Pearson correlation graphs of selected spliceosome components. Pearson correlation score (R) and p-value shown on-figure. Regression-line confidence interval shown in blue. Data points represent the mean of n=3 observations, matched by experimental group (i.e. Mock/RSV ± ZL0454).
